# Canine oral squamous cell carcinoma as a spontaneous, translational model for radiation and immunology research

**DOI:** 10.3389/fonc.2022.1033704

**Published:** 2023-01-09

**Authors:** Mary-Keara Boss, Lauren G. Harrison, Alexandra Gold, Sana D. Karam, Daniel P. Regan

**Affiliations:** ^1^ Flint Animal Cancer Center, Colorado State University, Fort Collins, CO, United States; ^2^ Department of Environmental and Radiological Health Sciences, Colorado State University, Fort Collins, CO, United States; ^3^ Department of Microbiology, Immunology, and Pathology, Colorado State University, Fort Collins, CO, United States; ^4^ Department of Clinical Sciences, Colorado State University, Fort Collins, CO, United States; ^5^ Department of Radiation Oncology, University of Colorado Anschutz Medical Campus, Aurora, CO, United States

**Keywords:** head and neck cancer, oral carcinoma, dog, radiation, immunology

## Abstract

**Introduction:**

Improving outcomes for oral squamous cell carcinoma (OSCC) patients has been hindered by a lack of effective predictive animal models. Spontaneously occurring canine OSCC could help fill this gap. The objective of this study was to characterize the immune landscape of canine OSCC to advance understanding of how dogs could serve as a surrogate for human OSCC.

**Methods/Results:**

Canine OSCC contains a heterogenous tumor immune microenvironment. CD3+ T cells were the predominant tumor infiltrating immune cell population; however, there was a wide range CD3+ T cell density across samples. The most common CD3+ T cell micro-anatomical distribution was defined as “pre-existing immunity”, but the remaining 20% of tumors were characterized as “immunologically ignorant” or “excluded infiltrates” patterns. When compared to normal oral mucosa, the tumor gene expression pattern suggests that canine OSCC microenvironment is highly inflamed and characterized by the presence of an anti-tumor immune response dominated by cytotoxic\effector T cells and NK cells (CD8a, GZMA, OX40, and HLA-A); however, overexpression of genes associated with effector T cell exhaustion and microenvironmental immunosuppression was also identified (PD-1, LAG3, CXCL2). Correlations between CD3+ T cell density and immune gene expression revealed key genes associated with cytotoxic anti-tumor T cell responses (GZMA, GZMB, PRF1), co-stimulation of T cells (CD27, CD28, ICOS), and other immune processes, including Type I IFN response (TNF, TNFSF10), and T cell exhaustion (CTLA4, PD-1). CD3+ T cell density in canine OSCC was significantly correlated with a cytolytic activity score (mean PRF1 and GZMA expression), suggestive of active effector CD8 T cell function. CD204+ macrophages were the second most abundant tumor infiltrating immune cell, and when comparing to normal oral mucosa, two differently expressed genes linked to tumor associated macrophages and myeloid derived suppressor cells (MDSC) were identified: CXCL2, CD70. Overexpression of CXCL2 was also identified in canine OSCC “T cell-high” tumors compared to “T cell-low” tumors.

**Discussion:**

This study identified actionable immunotherapy targets which could inform future comparative oncology trials in canine OSCC: CTLA-4, PD-1, CXCL2. These data provide a good first step towards utilizing spontaneous canine OSCC as a comparative model for human OSCC radiation and immuno-oncology research.

## 1 Introduction

Head and neck cancer comprises various malignancies that develop in or around the oral cavity, throat, larynx, or sinonasal cavity, and collectively, represents the sixth most prevalent cancer worldwide (1). Most head and neck cancers are squamous cell carcinomas (HNSCC) and most are of the oral cavity (OSCC). The 5-year overall survival rate for HNSCC is 40-50%, with a worse prognosis for patients with advanced disease (2, 3), which represents more than half of initially diagnosed HNSCC patients (4). The conventional standard-of-care for patients with advanced HNSCC is either chemoradiation or aggressive surgical excision of the tumor followed by 6 to 7 weeks of radiation therapy with or without chemotherapy (3). Dysregulated tumor cell proliferation has been shown to associate with treatment resistance [reviewed in (5–7)], and immune evasion within the HNSCC tumor microenvironment has also been shown to correlate with recurrence and metastasis (8–11). Targeted therapies aimed at countering proliferative signaling have failed, and new efforts underway to integrate immunotherapy to improve treatment outcomes have been met with limited success (12, 13).

While progress has been made in understanding the biology of HNSCC and OSCC, translating traditional laboratory research findings into clinical success has been overall slow and disappointing. In particular, a challenge to studying human papilloma virus (HPV)-negative HNSCC is the lack of effective predictive animal models. Improving cancer treatment outcomes for patients with advanced HPV-negative HNSCC relies on the availability of appropriate preclinical animal models. Comparative oncology research can fill an important gap between pre-clinical rodent and human studies, ultimately providing valuable, predictive, translational results for human cancer patients (14). Companion animals naturally develop a variety of cancers, many of which have been demonstrated to have significant biological overlap with their human counterparts, including shared genomic aberrations and molecular drivers, and a comparable clinical course of disease (14). Pets receive state-of-the-art medical care, which can include experimental therapeutics and novel technologies, offering an important opportunity for clinical cancer discovery (15). The National Cancer Institute has developed the Comparative Oncology Program to promote this area of increased research interest.

Spontaneously occurring canine OSCC share several characteristics with human OSCC, which makes it a relevant preclinical model. Canine OSCC is a locally invasive tumor, commonly invading bone, with the potential for metastasis to regional lymph nodes and distant sites late in the course of disease (16, 17). Additionally, it has been shown that spontaneously occurring carcinoma of the head and neck in dogs also represents the human counterpart at the molecular level with analogous genomic copy number abnormalities and mutational landscapes, alteration of known HNSCC genes and pathways, and comparably extensive intertumor heterogeneity (17). Notably, canine OSCC develops naturally in a setting of an intact immune system. The major cellular subsets of the dog immune system have been characterized and there is significant homology to humans, opening the door for translational cancer immunology research utilizing comparative oncology trials (18).

Recently, there have been investigations into treating human and canine HNSCC patients with stereotactic body radiotherapy (SBRT). SBRT, which allows delivery of high dose, high precision radiation in a few fractions, is a radiotherapy technique that can be used to treat HNSCC patients (19–24). Another positive aspect of SBRT treatment for HNSCC is reduced overall treatment time, and a decreased probability of accelerated repopulation (25), a biological phenomenon long associated with radio-resistance of HNSCC (26). Regarding tumor immunity, evidence exists that SBRT may be a more potent activator of anti-tumor immune responses compared to conventional radiotherapy (27). Emerging preclinical and clinical data suggest SBRT combined with immunotherapy has the potential to convert immunologically “cold” tumors into “hot” tumors by a combination of distinct mechanisms including increasing tumor immunogenicity *via* the upregulation of tumor antigen expression, antigen processing, major histocompatibility (MHC) molecule expression, and costimulatory signals; overcoming an immunosuppressive tumor microenvironment by shifting the cytokine balance in favor of immunostimulation; and recruiting antigen-presenting and immune effector cells to the tumor microenvironment (28–34). The ability to effectively harness the therapeutic benefits of radiotherapy and anti-tumor immunity could translate to considerable clinical improvements for HNSCC patients with advanced disease. However, the current reality is that combination radiation therapy (RT) and immunotherapy, despite demonstrable promise in different cancers, carries a poor overall response rate, particularly in a disease such as advanced HNSCC (35).

Thus, continued efforts are necessary to both identify and better optimize novel approaches to combining RT and immunotherapy in order to increase response rates and therapeutic outcomes for cancer patients, including those with advanced HNSCC (12). Pre-clinical vetting of novel radio-immunotherapy combinations in trials involving canine OSCC patients could accelerate translational preclinical research and bridge the gap between mechanistic rodent-based research and prolonged human clinical trials. Determining the safety and efficacy of novel RT and immunotherapy combinations in dogs with naturally occurring head and neck cancer could serve as a clinical surrogate with high predictability to inform how best to time, sequence, and dose these combination treatments, as well as identify more robust biomarkers for stratification of those patients most likely to response to these therapies.

With that goal in mind, the objective of this study was to characterize the baseline immune landscape of spontaneous canine OSCC tumors, as a critical and necessary first step towards utilizing dogs as a surrogate for human OSCC. We provide the first characterization of the immunogenomic landscape of canine OSCC and demonstrate the presence of relevant immunotherapeutic targets that could be translationally valuable for future comparative radio-immunotherapy trials in dogs with spontaneous OSCC.

## 2 Materials and methods

### 2.1 Patient samples

Archived frozen and formalin-fixed paraffin-embedded (FFPE) canine OSCC and normal mucosal tissue samples were obtained from the Flint Animal Cancer Center tissue biorepository and Colorado State University Veterinary Diagnostic Laboratory.

### 2.2 Histology and immunohistochemistry

Hematoxylin and eosin slides were reviewed by a board-certified pathologist (DPR) to confirm diagnosis and the presence of adequate viable tumor tissue prior to immunohistochemical labeling. Immunohistochemistry was performed using a Leica Bond Max autostainer (Leica Biosystems Inc.), with the following panel of previously published canine cross-reactive primary antibodies directed against the following antigens/cell types, at the listed concentrations: monoclonal mouse anti-human CD3 (pan T lymphocyte marker; Leica, clone LN10; 10 μg/mL), monoclonal mouse anti-human CD204 (macrophages; TransGenic Inc., clone SRA-E5; 1.25 μg/mL), mouse monoclonal anti-human CD79a (B lymphocytes; Abcam, clone HM57; 10 μg/mL), and mouse monoclonal anti-human FoxP3 (regulatory T cells; ThermoFisher, clone eBio7979; 5 μg/mL). Deparaffinization and rehydration was performed on the Bond autostainer using a series of xylenes and graded ethanols. Antigen retrieval was performed using either: Leica Epitope Retrieval 2 (Tris-EDTA buffer, pH 9; CD3, CD204, FoxP3), or Leica Epitope Retrieval 1 (Citrate buffer, pH 6; CD79a), both for 20 min at 100 °C. Detection was performed with PowerVision IHC detection systems (Leica Biosystems, Inc.), using a polymeric horseradish peroxidase anti-mouse IgG (CD204) and Bond Polymer Refine DAB chromogen, with routine hematoxylin counterstain.

For quantitative analysis of tumor-infiltrating leukocyte (TIL) density, whole slide brightfield images of IHC stained slides were digitally captured using an Olympus IX83 microscope with the Olympus SC30 camera at 10x magnification and fixed exposure times for all samples. Quantitative image analysis was performed using ImageJ software (National Institutes of Health, NIH). Brightfield images were converted to gray scale images for analysis. Tumor tissue regions-of-interest (ROIs) were segmented from adjacent normal tissue by manual annotation in ImageJ in blinded fashion, with annotation accuracy confirmed by a board-certified veterinary pathologist. Following determination of the ROI, positively labeled immune cells were quantified using the color deconvolution algorithm. Briefly, a positive pixel threshold for all immune cell markers was determined visually by a veterinary pathologist using appropriate isotype-stained control slides. Images were subjected to color deconvolution, followed by uniform automated application of this positivity threshold to all images. Following analysis, positive pixel masks of each image were blindly evaluated by a pathologist to ensure feature selection accuracy. Data was analyzed and the number of infiltrating immune cells was expressed as a percentage of total tumor tissue area.

### 2.3 Histological characterization of tumor immunity pattern

Whole slide digital images of all CD3+ immunolabeled tumors slides were blindly qualitatively scored (by LH, MKB, and DPR) to characterize the distribution pattern of tumor-infiltrating T cells (“pre-existing immunity”, “excluded infiltrate”, or “immunologically ignorant”), according to Hedge, et al (36).

### 2.4 Nanostring gene expression analysis

RNA was extracted from archived frozen and FFPE tumor and normal tissues using Qiagen RNeasy and Qiagen RNeasy FFPE kits, respectively. For the FFPE samples, five to seven tissue sections (5-10 µm thick) were cut from each block and pooled for RNA extraction. RNA was extracted from flash-frozen lymph node tumor tissues using the RNeasy Plus Mini Kit (QIAGEN) following manufacturer protocol. Depending on starting material, samples were eluted in 30-50uL and were initially checked for quantity and purity on a Nanodrop ND-1000 Spectrophotometer (Thermo Fisher) prior to being stored at -80c until further processing. Samples were additionally quantity and quality checked using the RNA High Sensitivity assays on the Qubit 2.0 Fluorometer (Invitrogen/LifeTechnologies) and 5200 Fragment Analyzer Automated CE System (Agilent), respectively. NanoString gene expression analysis was performed using a custom-designed 48 gene canine immune panel derived from Rooney et al (37). The genes included in this panel are listed in **Supplementary Table 1**. Nanostring analysis was performed with the nCounter Analysis FLEX system at the University of Arizona Genetics Core. Gene expression count data was analyzed *via* nSolver software.

### 2.5 Statistical analyses

All statistical analyses were performed using commercial software (Prism 8; GraphPad Software, Inc.). Continuous data are expressed as means ± standard deviation. For assessment of correlations between immune cell IHC scores and Nanostring gene expression data, tumor samples were stratified as either high (“immunologically hot”) or low (“immunologically cold”) immune cell infiltration according to median dichotomization of percent density. IHC scores and Nanostring gene counts were then log-transformed, and correlations assessed by Spearman non-parametric rank correlation with correlations containing r values >0.5 or <-0.5. For all analyses, the statistical significance level was defined as alpha < 0.05.

## 3 Results

### 3.1 Patient characteristics

Tissue samples from 33 dogs were analyzed, which included 34 tumor samples and 2 normal mucosal samples, with one dog providing both tumor and normal mucosal samples. Breeds of the dogs consisted of mixed breed (n=11, 33%), Labrador retriever (n=4, 12%), golden retriever (n=3, 9%), and one each of the following: papillon, boxer, American Eskimo dog, Boston terrier, pug, Doberman pinscher, Jack Russell terrier, Siberian husky, vizla, shih tzu, German shepherd, Australian shepherd, cocker spaniel, west highland white terrier, and standard poodle. Within this group, there was one intact male (3%), one intact female (3%), 15 spayed females (46%), and 16 neutered male dogs (48%). The median age of the dogs at diagnosis was 10 years old (range 5 months – 14 years; not reported for two dogs).

### 3.2 Immunohistochemical analysis of tumor-infiltrating leukocytes

**Figure 1:**
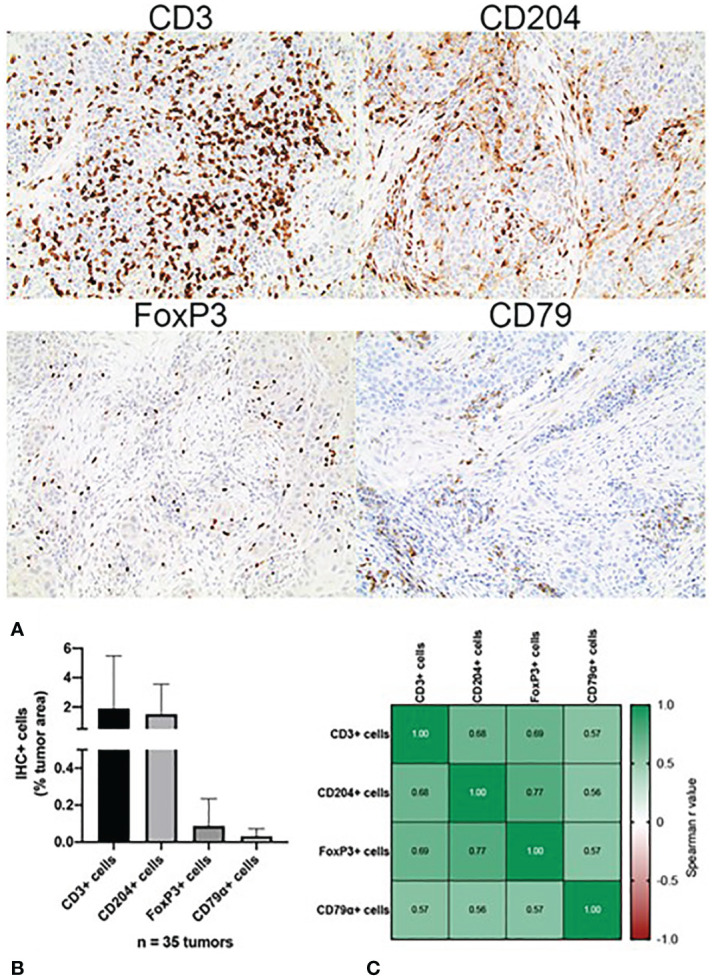
Immunohistochemical characterization of the immune landscape of canine OSCC. **(A)** Representative photomicrographs of CD3+, CD204+, FoxP3+ and CD79a+ tumor infiltrating leukocytes. 10x magnification. **(B)** Quantification of tumor-infiltrating leukocyte density according to cell type, as determined by quantitative whole slide image analysis. Data represent mean +/- S.D. **(C)** Correlation matrix of Spearman r values demonstrating association between the densities of each tumor-infiltrating leukocyte subset.

Tumoral immune cell infiltration was quantified *via* IHC in all (n=34) canine OSCC samples. The density of tumor infiltrating CD3+ T cells, expressed as percentage of total tumor area, ranged from 0.001% to 19.31%. The density of CD204+ macrophages ranged from 0.002% to 7.02% while the density of CD79a+ B cells ranged from 0% to 0.155%, and the density of FoxP3+ Treg cells ranged from 0% to 0.504%. CD3+ T cells accounted for the highest mean percentage of tumor-infiltrating leukocytes (1.57% +/- 3.68), followed by macrophages (0.83% +/- 1.45, regulatory T cells (0.05% +/- 0.10), and B cells (0.02% +/- 0.04), respectively (**Figures 1A, B**). With regard to any co-dependent relationships between infiltrating immune cell types, significant correlations were found between the density of: CD3+ T cells with CD204+ macrophages (r=0.681, p=7e^-6^), FoxP3+ cells (r=0.692, p=4e^-6^), and CD79a+ cells (r=0.572, p=3.4e^-4^); CD204+ macrophages and FoxP3 (r=0.767, p=7.9e^-8^) and Cd79a+ cells (r=0.563, p=0.0004); and FoxP3+ cells and CD79a+ cells (r=0.565, p=0.0004) (**Figure 1C**).

**Figure 2:**
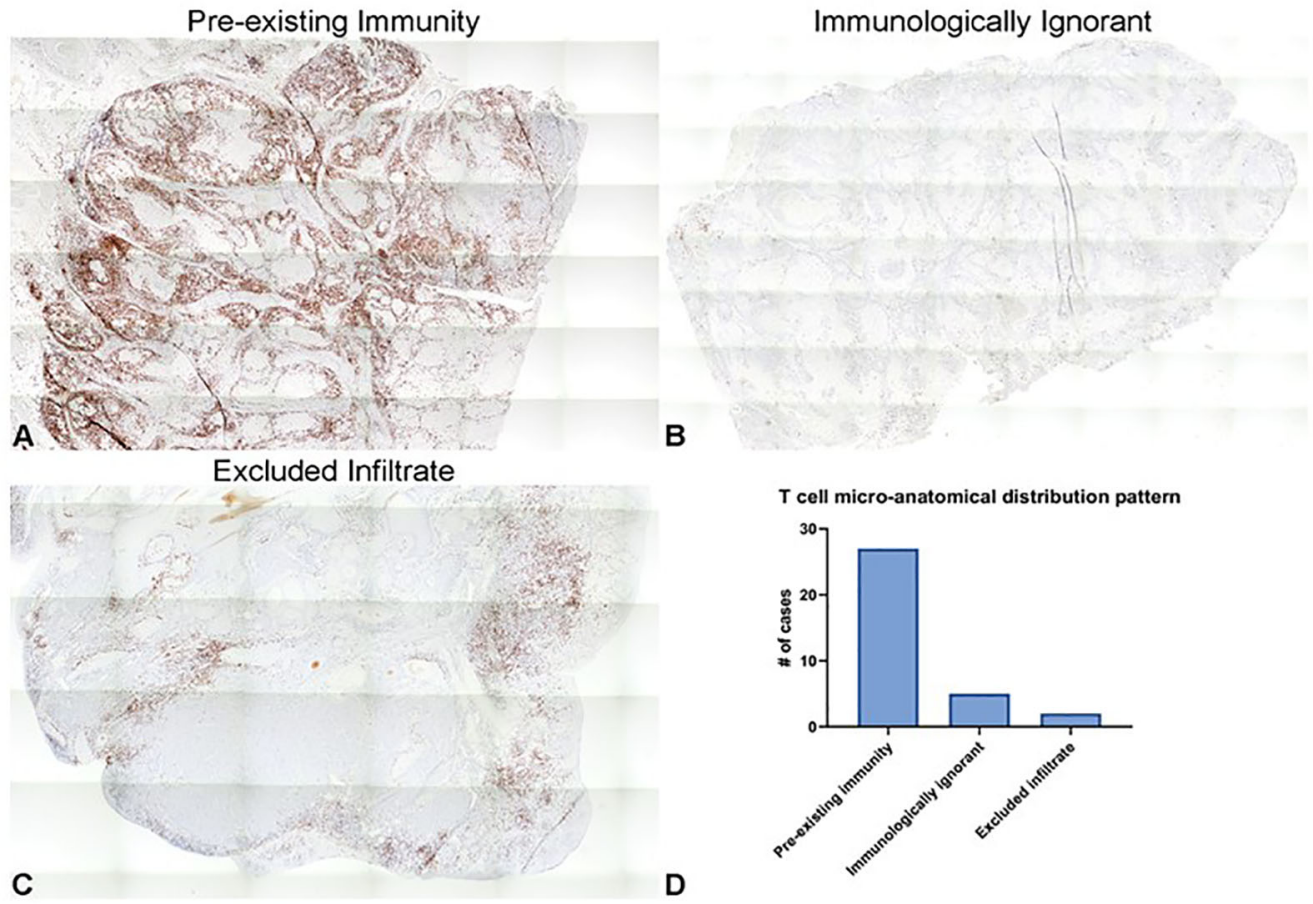
Distribution patterns of CD3+ tumor-infiltrating T cells in canine OSCC represent those observed in human cancers. **(A–C)** Representative photomicrographs of the three different CD3+ cellular infiltration patterns observed in our subset of cases, including “pre-existing immunity” **(A)**, “immunologically ignorant” **(B)**, and “excluded infiltrate” **(C)**. 5x magnification. **(D)** Quantification of the number of tumor samples characterized as each of the relative densities of T cell micro-anatomical distribution patterns.

CD3+ T cell micro-anatomical distribution within tumors was qualitatively analyzed according to those patterns described by Hegde et al (36). Specifically, the three CD3+ labeling patterns consist of “pre-existing immunity” which is considered highly inflamed, “excluded infiltrate” which is considered immunosuppressive, and “immunologically ignorant” which is considered non-inflamed (36). Within the tumor samples analyzed, 27 cases (77%) were characterized as “preexisting immunity”, five (14%) as “immunologically ignorant”, and two (6%) as “excluded infiltrates” (**Figure 2**). Representative images from three of the tumors, one of each pattern, are shown in **Figures 2A–C**.

### 3.3 Immune-related gene expression profiling of tumors

**Figure 3:**
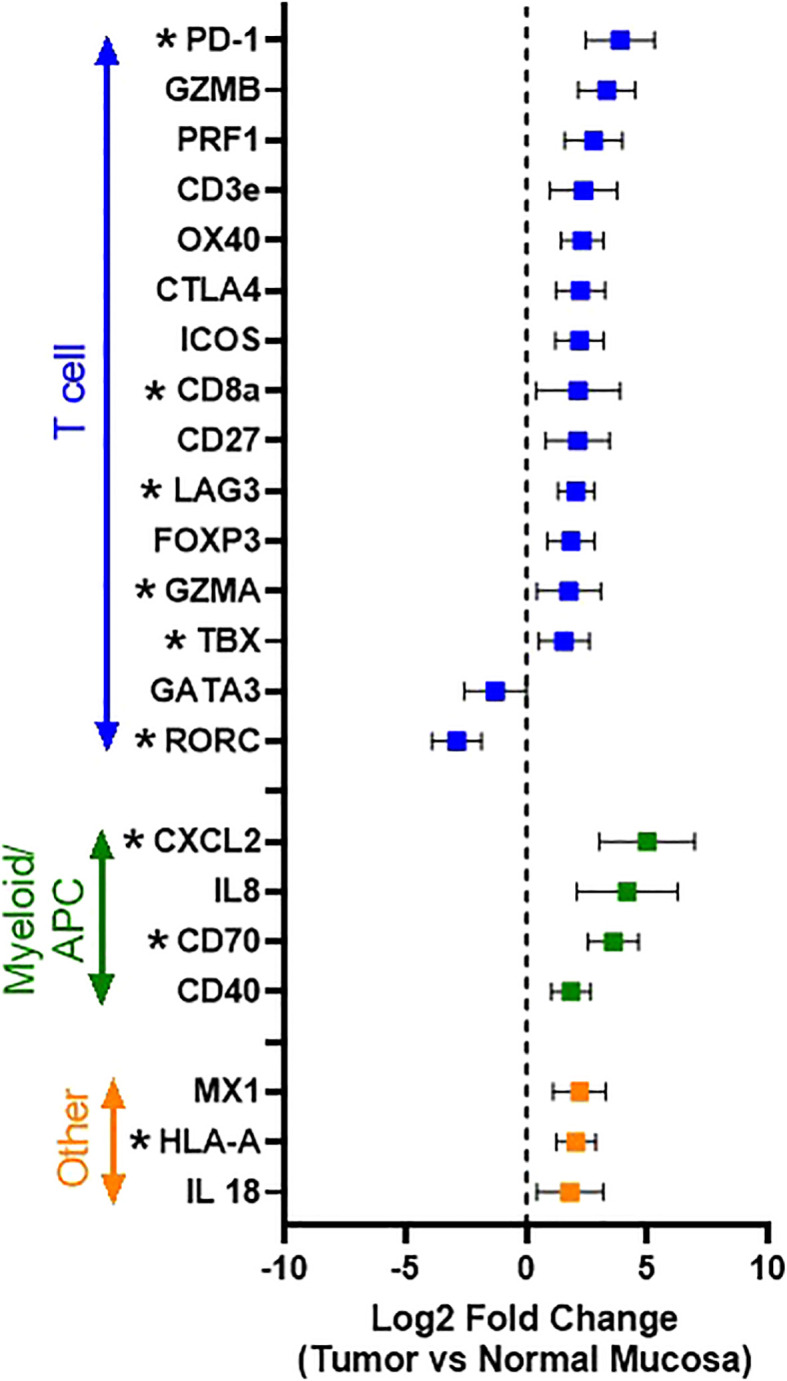
Immune gene expression patterns in canine OSCC. Log2 fold change forest plot of differentially expressed genes with a log2 fold change >1 or < -1 between tumors (n=34 and normal oral mucosa (n=2). Genes are groups according to their immune cell type and/or function. Data represents mean +/- S.D. * indicates significantly differentially expressed genes with an FDR-adjusted p-value <0.05.

Comparing all tumors (n = 32) to normal oral mucosa (n=2), 22 differentially expressed genes with Log2 fold-change of >1 or <-1 were identified, and, of these, 11 genes reached statistical significance based on an FDR-adjusted p value of <0.05. The following genes were significantly overexpressed in tumor samples: CD8a (p<0.0001), GZMA (p=0.0022), IFNγ (p<0.0001), PD-1 (p<0.0001), TBX (p<0.0001), CXCL2 (p=0.0005), CD70 (p<0.0001), HLA-A (p<0.0001), LAG3 (p<0.0001), while the expression of RORC (p<0.0001), and CLEC4C (p<0.0001) were significantly downregulated in tumor samples (**Figure 3**). Broadly, this pattern of tumor immune gene expression, in conjunction with the CD3 immunolabeling results, suggests that the canine OSCC microenvironment is highly inflamed and primarily characterized by the presence of a pre-existing anti-tumor immune response dominated by cytotoxic\effector T cells and NK cells, as evidenced by increased expression of CD8a, GZMA, OX40, and HLA-A. However, overexpression of genes associated with effector T cell exhaustion and microenvironmental immunosuppression (PD-1, LAG3, CXCL2) was also identified.

**Figure 4:**
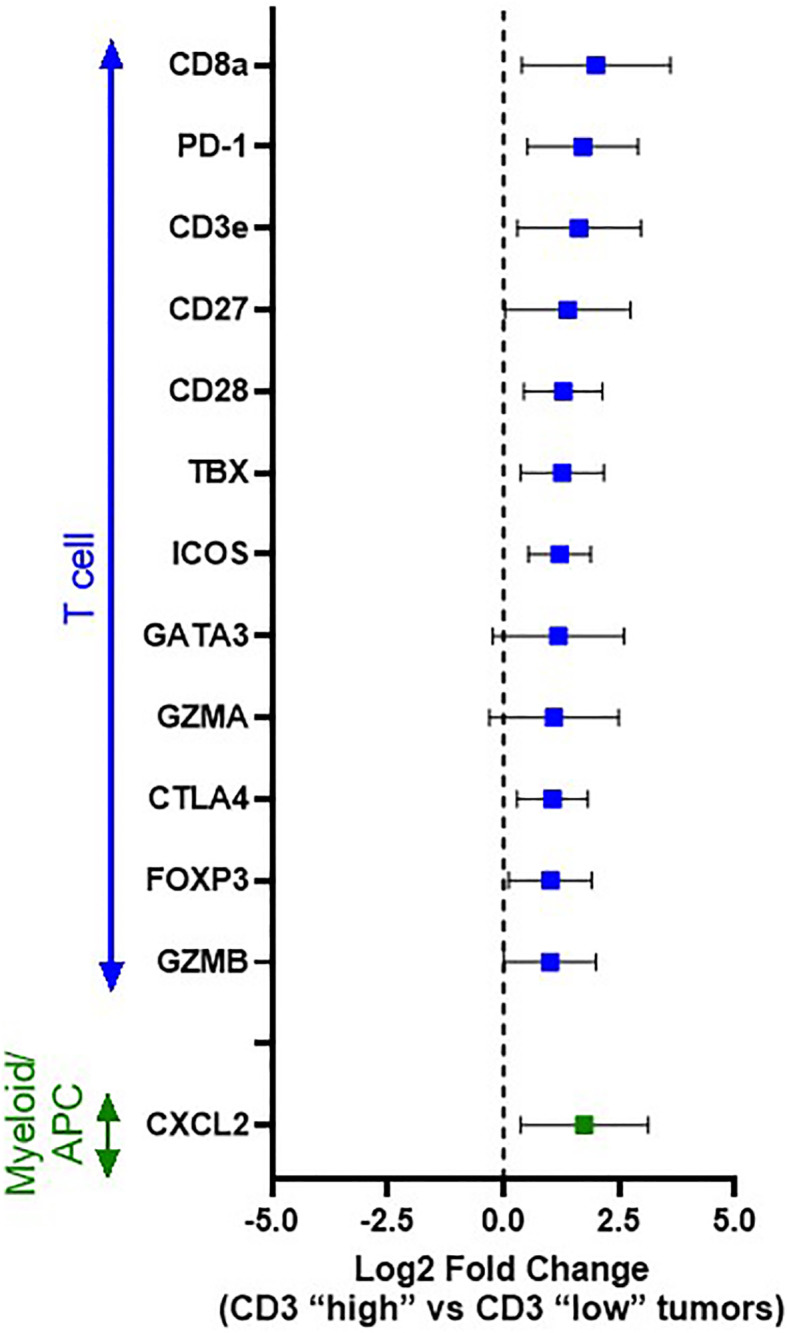
Differential immune gene expression between immunologically “hot” vs. “cold” canine OSCC. Log2 fold change forest plots of differentially expressed genes with an FDR q value <0.05 between CD3 “high” (n=17) vs CD3 “low tumors” (n=17). Tumors were stratified into CD3 “high” vs “low” groups based on median dichotimization of IHC density. Genes are grouped according to immune cell type/function. Data represent mean +/- S.D.

Thus, to gain further insight into those immune genes associated with a heightened T cell response in canine OSCC, differential gene expression analysis was performed comparing those tumors with “high T cell infiltration” versus “low T cell infiltration” based on median dichotomization of CD3+ cell density according to our immunohistochemical profiling (**Figure 4**). Twenty-one genes with significantly different expression were identified (**Supplemental Table 1**); however, when considering those genes with Log2 fold-change of >1 or <-1, 13 genes were revealed as significantly differentially expressed between “high” versus “low” T-cell infiltrated tumors. Notably, CD8a was significantly upregulated (p=0.0003), demonstrating that these CD3+ T cells are primarily of a cytotoxic phenotype, consistent with the presence of a pre-existing immune response. Other significantly up-regulated genes in “T cell high” tumors included many immune checkpoint molecules, including both co-stimulatory molecules (ICOS (p=0.0001) and CD28 (p=0.0002), as well as co-inhibitory molecules associated with an exhausted phenotype in human cancer patients (PD-1 (p=0.0001), CTLA-4 (p=0.0014) [reviewed in Chen, et al. (13)].

Interestingly, another significantly up-regulated gene in “T cell high” tumors was CXCL2 (p=0.0081), a chemokine known to play a significant role in myeloid-derived suppressor cell (MDSC) recruitment and differentiation [reviewed in Safarzadeh et al. (38)], suggesting a potential role for tumor-associated macrophages (TAMs)/MDSCs in mediating the expression of the T cell exhaustion markers (PD-1) observed to be upregulated in our T cell high tumors. Thus, based on this result, we also performed differential gene expression analysis between those tumor samples with a high infiltration of CD204+ TAMs versus low CD204+ density, again based on median dichotomization of CD204+ density as determined by our immunohistochemical analysis; however, the analysis did not reveal any significantly differentially expressed genes.

### 3.4 Correlation between tumor-infiltrating immune cell density and immune gene expression

**Figure 5:**
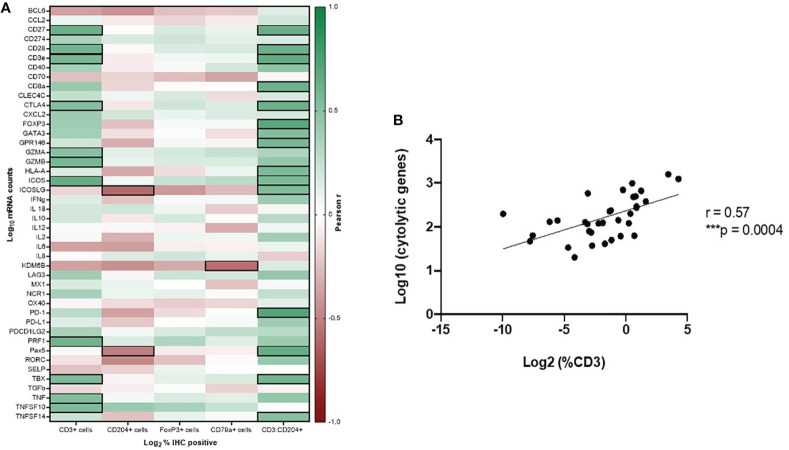
Gene expression profiling to determine immune function of tumor-infiltrating leukocyte subsets in canine OSCC. **(A)** Pearson correlation matrix of immune cell densities of all analyzed cell types, as determined by IHC, with Nanostring gene expression data. Black boxes denote statistically significant (p < 0.05) correlations with r values > 0.5 or < -0.5. **(B)** Spearman correlation plot of CD3-IHC+ cell density with cytolytic activity score (mean perforin and granzyme expression values).

Statistically significant correlations were found between CD3+ T cell infiltration as determined by IHC and expression of 23 immune genes in our panel, with 11 of these genes having Pearson r values > 0.5 or < -0.5. These included key genes associated with cytotoxic anti-tumor T cell responses (GZMA, GZMB, PRF1), co-stimulation of T cells (CD27, CD28, ICOS), and genes associated with other immune processes, including Type I IFN response (TNF, TNFSF10), as well as T cell exhaustion (CTLA4). CD204+ IHC cell density was significantly correlated with expression of 9 genes in our panel, with 2 genes having Pearson r values > 0.5 or < -0.5, which included decreased expression of ICOSLG and Pax5. Correlation of CD204+ TAM infiltration and decreased expression of these genes further suggests evidence of microenvironmental immune suppression in these samples. ICOSLG is the ligand which serves as the specific receptor for ICOS, as the ICOS/ICOSLG axis is associated with regulating T cell-antigen presenting cell interactions (39, 40). FoxP3+ cell density as determined by IHC was significantly correlated with decreased expression of ICOSLG, but with an r value < -0.5. Cd79a+ cell density was correlated with decreased expression of KDM6B, a gene associated with the generation of effector CD8+ T cells (41, 42). Finally, statistically significant correlations were found between the ratio of CD3+:CD204+ cell density as determined by IHC and expression of 25 genes, with 15 of these genes having Pearson r values > 0.5 or < -0.5. These included genes consistent with pro-inflammatory immune responses (CD27, CD28, CD3e, CD8a, ICOS, ICOSLG, Pax5, TBX, GATA3, HLA-A, TNFSF14, GPR146), but also immune suppressive influences in the tumor immune microenvironment (CTLA-4, FoxP3, PD-1). These correlations are shown in **Figure 5A** and **Table S3**.

Additionally, according to Rooney et al. (37), we calculated a cytolytic activity score (mean PRF1 and GZMA expression) for each tumor sample and assessed its correlation with CD3+, CD79a+, CD204+, and FoxP3+ cell density as determined by IHC. CD3+ T cell density by IHC analysis was strongly correlated with cytolytic activity score (r = 0.57, p = 0.0004) (**Figure 5B**).

## 4 Discussion

The objective of this study was to characterize the baseline immune landscape of spontaneous canine OSCC tumors as a critical initial step towards utilizing dogs with this naturally occurring cancer as a surrogate for human OSCC. The tumor microenvironment of human HNSCC, of which OSCC is the most common, consists of multiple subsets of infiltrating immune cells that interact with tumor cells and each other through various networks. There is a spectrum as to the degree of inflammation in the tumor immune microenvironment across the subsets of human HNSCC, ranging from immune-cold to inflamed phenotypes [reviewed in (12, 13, 43)].

As we have documented in this study, canine OSCC also contains a heterogenous tumor immune microenvironment. CD3+ T cells were the predominant tumor infiltrating immune cell population in the tumor samples; however, there was a wide range in the density of CD3+ T cells across the samples, from 0.001% to 19.31% cells of the total tumor area. Further, upon histological characterization of the tumor immunity pattern, the CD3+ T cell micro-anatomical distribution associated with most tumors was characterized as “pre-existing immunity”, but the remaining 20% of tumor samples were characterized as either “immunologically ignorant” or “excluded infiltrates” patterns. When the immune-related gene expression profile of the tumors was compared to normal oral mucosa, the pattern of tumor immune gene expression suggests that the canine OSCC microenvironment is highly inflamed and primarily characterized by the presence of an anti-tumor immune response dominated by cytotoxic\effector T cells and NK cells (CD8a, GZMA, OX40, and HLA-A); however, overexpression of genes associated with effector T cell exhaustion and microenvironmental immunosuppression was also identified (PD-1, LAG3, CXCL2).

We propose that canine OSCC could serve as a relevant translational animal model for comparative investigations into the biology of HNSCC and OSCC. Alcohol and tobacco use (smokers drinkers, SD) is associated with the development of human HNSCC; while these predisposing factors cannot be investigated translationally in companion dogs, the canine model may still be valuable for studying never-smoker and never-drinker (NSND) patients with OSCC, which comprise 10-15% of cases (44, 45). Oncogenic human papillomavirus (HPV) infection is also a risk factor associated with oral and oropharyngeal HNSCC. HPV-positive and HPV negative HNSCC are two distinct biological entities with different clinical behaviors and outcomes; however, the basic immunobiology of HNSCC, particularly differences between HPV-positive and HPV-negative immune tumor microenvironments, remains largely undefined and an area of active research (45–47). Dogs can be infected with canine papillomavirus (CPV), but rarely do these infections persist and progress beyond papillomas to malignant cancer. CPV2 has been associated with metastatic cutaneous SCC in a research colony of dogs with X-linked severe combined immunodeficiency (48), and there have been rare reports of cancer, including OSCC, associated with other CPV (49–53). Recently, integration of CPV16 into the host genome was reported, having investigated the viral genome from a viral plaque that progressed to metastatic SCC in a dog, raising the possibility that CPV16 may be a potential canine high-risk papillomavirus type (54). While most canine HNSCC are considered CPV-negative, there may be a role for future comparative oncology research investigating similarities and differences in the TME and therapeutic responses in human and canine papillomavirus-negative versus positive HNSCC.

The human HNSCC tumor microenvironment is composed of multiple different subsets of stromal and innate and adaptive immune cells including cancer-associated fibroblasts, T cells, B cells, neutrophils, macrophages, MDSCs, natural killer (NK) cells and mast cells (13, 55, 56). In one study of OSCC patient tumor samples, it was observed that the majority of intratumor tumor-infiltrating lymphocytes (TILs) were CD8+ T cells (57); this is consistent with the findings in our canine OSCC study where high CD3+T cell-infiltrated tumors had significant overexpression of CD8a. CD8a was significantly upregulated gene in “T-cell high” versus “T-cell low” tumors, suggesting that the observed CD3+ T cell infiltrates are primarily of a cytotoxic phenotype, consistent with the presence of a pre-existing immune response. We also evaluated correlations between CD3+ T cell density and immune gene expression and key genes associated with cytotoxic anti-tumor T cell responses (GZMA, GZMB, PRF1), co-stimulation of T cells (CD27, CD28, ICOS), and genes associated with other immune processes, including Type I IFN response (TNF, TNFSF10), as well as T cell exhaustion (CTLA4), were identified. Finally, CD3+ T cell density in canine OSCC was significantly correlated with a previously reported cytolytic activity score (mean PRF1 and GZMA expression), suggestive of active effector CD8 T cell function in the tumor microenvironment.

Exhausted and dysfunctional TILs in human HNSCC cases have been characterized by the upregulation of several inhibitory checkpoint molecules, including PD-1, LAG-3, TIM-3, and CTLA-4. In one study, CD8+ TILs of human HNSCC were found to express PD-1 in 96% of evaluated samples (58). Evaluation of HPV-negative HNSCC samples revealed 40% of cases exhibited PD-L1 positivity associated with tumor cells, lymphocytes, and macrophages, and approximately 50% of these samples also showed positive PD-1 expression (46). In that study, PD-L1 and PD-1 positivity correlated with a high density of both CD8+ and FOXP3+ TILs (46). In our study, we found that canine OSCC tumors which were heavily infiltrated with T cells had up-regulation of immune checkpoint molecule genes including both co-stimulatory molecules (ICOS, CD28), as well as co-inhibitory molecules known to be associated with an exhausted immune phenotype (PD-1, CTLA-4).

Tumor infiltrating T regulatory (Treg) cells also influence the immune microenvironment of HNSCC. In HNSCC, Tregs have been associated with immunosuppression, anti-inflammatory cytokine production, and therapeutic resistance (33, 59–61); however, increased densities of FoxP3+ Tregs in HNSCC has also been linked with improved therapeutic outcomes for treated patients (62–64). As Tregs serve to regulate excessive immune reactivity to prevent auto-immunity, it is theorized that the presence of high densities of FoxP3+ Tregs in HNSCC could indicate an active, robust antitumor immune response, contributing to improved tumor control (13). This is an area of active research, and perhaps canine OSCC may serve a role in understanding the tumor microenvironmental effects of Tregs. Infiltrating FoxP3+ cells were identified in canine OSCC tumors in this study and the density of these cells significantly correlated with the densities of all other evaluated infiltrating immune cells (CD3+ T cells, CD204+ macrophages, and CD79a+ B cells). Further, overexpression of FOXP3 was identified in our CD3+ T cell-high tumors as compared to T cell-low tumors, and its expression was also significantly positively correlated with the ratio of CD3+:CD204+ cells, suggesting their presence may be an indicator of an overall inflamed tumor microenvironment in canine OSCC.

Myeloid cells represent another heterogenous cell population with significant influences on the tumor microenvironment and anti-tumor immunity. Tumor-associated macrophages (TAMs) have been shown to be enriched in human HNSCC tumors, and increased infiltrating TAM density has been shown to correlate with advanced stage of disease (65). Additionally, the clinical relevance of myeloid-derived suppressor cell (MDSC) subsets is also under investigation in human HNSCC patients, and strong correlations of MDSC subsets with treatment outcome have been made recently (66). Although TAMs and MDSCs are regarded as separate entities, the boundaries between them are not clearly demarcated and they share many characteristics (67, 68). In our study, immunohistochemistry identified CD204+ macrophages as the second most abundant tumor infiltrating immune cell. Moreover, when comparing canine OSCC tumor gene expression to normal oral mucosa, two differently expressed genes associated with the presence of TAMs and MDSCs were identified: CXCL2, CD70. Additionally, overexpression of CXCL2 was also identified in our canine OSCC “T cell-high” tumors compared to “T cell-low” tumors. CXCL2 is a chemokine known to play a significant role in myeloid-derived suppressor cell (MDSC) recruitment and differentiation (38). Thus, it is possible that this CD204+ macrophage infiltrate observed may be contributing to expression of the T cell exhaustion markers (PD-1, CTLA-4) observed to be significantly upregulated in our “T cell-high” tumors. We also found that CD204+ IHC cell density in canine OSCC was significantly correlated with decreased expression of ICOSLG and Pax5. Correlation of CD204+ TAM infiltration and decreased expression of these genes further suggests evidence of microenvironmental immune suppression in these samples. ICOSLG is the ligand which serves as the specific receptor for ICOS, as the ICOS/ICOSLG axis is associated with regulating T cell-antigen presenting cell interactions (39, 40), and Pax5 regulates B cell immunity *via* promoting development and maturation of B cells (69).

Recently, a pan-cancer, T-cell-inflamed 18-gene signature indicative of a T-cell-activated tumor microenvironment was reported to be associated with response to PD-1 immune checkpoint blockade, including patients with HNSCC (70, 71). A canine anti-PD-1 therapeutic antibody has been developed and a pilot study investigating its safety and efficacy in treating dogs with a variety of spontaneous cancers demonstrated encouraging results, supporting further comparative investigation of this immunotherapy (72, 73). Moving forward, investigation of this human T-cell-inflamed gene expression profile in dogs with T cell targeted immunotherapies may also be useful. One limitation of our study is that the immune gene expression panel used was limited to a custom-designed 48 gene panel derived from Rooney et al. (37), and designed prior to the commercial release of the nCounter^®^ canine IO panel. Thus, not all of the 18 genes in the T-cell-inflamed gene expression profile were included in our panel, with those that were shared being only CD27, LAG3, and CD8a. In reviewing our results with respect to these shared genes, tumors compared to normal oral mucosa had significantly increased expression of CD8a and LAG3. Additionally, tumors with high T cell density also had significantly increased expression of CD27 and CD8a compared to those with low T cell density, and PD-1 was significantly upregulated gene in these “T cell high” tumors. These data suggest that investigation of the presence of a similar, pre-existing T cell inflamed gene signature as a correlate to response in trials of T cell targeted immunotherapies in dogs may be useful in determining how best to employ these newly developing canine specific immunotherapies.

This study also identified other actionable immunotherapy targets which could inform future comparative oncology trials in canine OSCC. Anti-CTLA-4 immunotherapy has been investigated for the treatment of HNSCC, with or without additional immune checkpoint inhibitors (anti-PD-1, anti-PDL1) or radiotherapy (reviewed in (12, 74). CTLA-4 was overexpressed in canine OSCC compared to oral mucosa, as well as in “T cell-high” compared to “T-cell low” tumors and correlated with the density of CD3+ cells and the ratio of CD3:CD204+ cells. A canine CTLA-4 monoclonal antibody has recently been developed for comparative oncology research (75); however, it has not been evaluated yet in canine cancer patients. Finally, clinical strategies aimed at targeting MDSC recruitment into the tumor microenvironment, such as *via* CXCL2 inhibition, may be critical to overcoming immunosuppression. The prognosis and therapeutic value of the CXC chemokine family, including CXCL2, in HNSCC is under investigation (76, 77). We found canine OSCC had increased CXCL2 expression compared to normal oral mucosa, and in “T-cell high” versus “T-cell low” tumor samples. Canine OSCC may contribute to investigations into whether inhibition of CXCL2 results in reduction in the recruitment and immune suppressive influence of MDSC in the OSCC microenvironment.

With respect to immune biomarkers of response for HNSCC undergoing treatment with radiotherapy or chemoradiotherapy, infiltration of CD3 lymphocytes in the tumor microenvironment and PD-L1 expression on tumor cells or immune cells have separately showed prognostic correlation with survival outcomes or response to radiotherapy (78, 79). Additionally, in multiple studies, retrospective analyses and meta-analyses have shown that the baseline circulatory neutrophil-to-lymphocyte ratio is a strong predictor of survival outcomes for chemoradiotherapy or radiotherapy treatment for patients with HNSCC (80–83). The degree to which radiotherapy, administered at various doses and with alternate timing and sequencing strategies, can generate a pro-inflammatory tumor microenvironment and increase T-cell inflamed gene expression in HNSCC is being investigated in pre-clinical animal experiments and ongoing clinical trials combining radiotherapy and immunotherapy (12). As the local, regional, and systemic immune effects of treatment can be similarly measured and characterized for canine OSCC patients, dogs may further contribute to the efforts underway to identify underlying immune biomarkers of response to treatment, including radiation and immunotherapy combinations. There are a number of published and ongoing efforts to explore novel immunotherapeutic approaches in canine cancer trials [reviewed in (18, 84)]; however, access to commonly used immunotherapy agents in human oncology, such as anti-PD-1, anti-PD-L1, and anti-CTLA-4 antibodies, remain limited in access in veterinary medicine, but with promise for availability in the future. As such, there has not yet been a clinical trial performed investigating responses to anti-PD-1, anti-PD-L1, or anti-CTLA-4 antibody immunotherapy, with or without radiotherapy, in canine OSCC, so comparisons of response with human OSCC or HNSCC are not possible at this time.

In conclusion, while these data provide a good first step towards utilizing spontaneous canine OSCC as a comparative model for human OSCC radiation and immuno-oncology research, there a few key limitations which if addressed in future investigations, would allow the field to fully realize the potential of this approach. The small number of tumor samples and immune genes evaluated in this study limits the impact of the observed correlates between cellular infiltrates and gene expression. Additional studies utilizing the larger 800 gene nCounter^®^ canine IO panel and expanding this data set with clinically annotated tumor samples in which TIL density and gene expression could be correlated with clinical outcome would be valuable. Moreover, pairing this immune microenvironment profiling with genomic analyses such as whole exome sequencing would also allow for comparative assessment of known human HNSCC tumor intrinsic molecular drivers, like EGFR, and correlation of the TME composition with both the presence of these molecular drivers as well as mutational burden. As studies are performed in canine OSCC, we will begin to understand more of the similarities and differences between human and canine OSCC within the TME, as well as regionally and distantly, and how tumor characteristics in each of these species correspond with response to treatment. Nonetheless, these results provide the first characterization of the immune landscape of canine oral carcinoma and demonstrate the presence of relevant immunotherapeutic targets that could be translationally valuable in the design of future comparative radio-immunotherapy trials in dogs.

## Data availability statement

The datasets presented in this study can be found in online repositories. The names of the repository/repositories and accession number(s) can be found below: Gene Expression Omnibus, GSE213726.

## Author contributions

Conceptualization of the study: M-KB, DR; investigation and analysis: M-KB, LH, AG, DR; writing – original draft preparation: M-KB; writing – review and editing: M-KB, SK, DR. All authors contributed to the article and approved the submitted version.
