# Supplementary material for: Canine oral squamous cell carcinoma as a spontaneous, translational model for radiation and immunology research
Source: Front Oncol. 2023 Jan 9;12:1033704. doi: 10.3389/fonc.2022.1033704 (PMC9868558; doi:10.3389/fonc.2022.1033704)
Supplement: Supplementary file 1 [file Table_1.docx]

| Gene Name |
| --- |
| BCL6 |
| CCL2 |
| CD27 |
| CD274 |
| CD28 |
| CD3e |
| CD40 |
| CD70 |
| CD8a |
| CLEC4C |
| CTLA4 |
| CXCL2 |
| FOXP3 |
| GATA3 |
| GPR146 |
| GZMA |
| GZMB |
| HLA-A |
| ICOS |
| ICOSLG |
| IFNγ |
| IL 18 |
| IL10 |
| IL12 |
| IL2 |
| IL6 |
| IL8 |
| KDM6B |
| LAG3 |
| MX1 |
| NCR1 |
| OX40 |
| PAX5 |
| PD-1 |
| PDCD1LG2 |
| PD-L1 |
| PRF1 |
| RORC |
| SELP |
| TBX |
| TGFβ |
| TNF |
| TNFSF10 |
| TNFSF14 |
| ALAS1 (housekeeping) |
| GUSB (housekeeping) |
| RPL30 (housekeeping) |
| SDHA (housekeeping) |

**Supplementary Table 1.** Customized Nanostring canine immune panel (48 genes)
