# Supplementary material for: Canine oral squamous cell carcinoma as a spontaneous, translational model for radiation and immunology research
Source: Front Oncol. 2023 Jan 9;12:1033704. doi: 10.3389/fonc.2022.1033704 (PMC9868558; doi:10.3389/fonc.2022.1033704)
Supplement: Supplementary file 2 [file Table_2.docx]

| **Gene** | **P value** | **Mean of CD3 "High"** | **Mean of CD3 "Low"** | **Difference** | **SE of difference** | **t ratio** | **df** | **q value** |
| --- | --- | --- | --- | --- | --- | --- | --- | --- |
| **CD27** | 0.001357 | 8.56 | 7.171 | 1.389 | 0.3886 | 3.574 | 26.85 | 0.002921 |
| **CD28** | 0.000197 | 8.408 | 7.116 | 1.292 | 0.3071 | 4.208 | 31.71 | 0.000995 |
| **CD3e** | 0.000242 | 11.1 | 9.467 | 1.636 | 0.3885 | 4.21 | 27.8 | 0.001015 |
| **CD40** | 0.000455 | 9.936 | 9.01 | 0.9261 | 0.2369 | 3.91 | 31.78 | 0.001433 |
| **CD8a** | 0.000311 | 10.37 | 8.364 | 2.004 | 0.4921 | 4.073 | 30.13 | 0.001118 |
| **CTLA4** | 0.00135 | 8.911 | 7.849 | 1.062 | 0.3009 | 3.53 | 30.3 | 0.002921 |
| **CXCL2** | 0.008107 | 13.85 | 12.1 | 1.75 | 0.6132 | 2.854 | 27.53 | 0.013619 |
| **FOXP3** | 0.001391 | 8.011 | 6.998 | 1.014 | 0.2891 | 3.506 | 31.43 | 0.002921 |
| **GATA3** | 0.00592 | 8.66 | 7.471 | 1.19 | 0.3962 | 3.002 | 25.53 | 0.010656 |
| **GZMA** | 0.013533 | 8.169 | 7.075 | 1.094 | 0.416 | 2.63 | 28.93 | 0.018245 |
| **GZMB** | 0.010979 | 9.366 | 8.359 | 1.006 | 0.3721 | 2.704 | 31.24 | 0.017292 |
| **HLA-A** | 0.013756 | 14.36 | 13.69 | 0.6743 | 0.2586 | 2.607 | 31.93 | 0.018245 |
| **ICOS** | 0.00013 | 10.28 | 9.064 | 1.216 | 0.2763 | 4.401 | 29.44 | 0.000818 |
| **IL10** | 0.038015 | 8.6 | 8.154 | 0.446 | 0.2056 | 2.17 | 30.29 | 0.045618 |
| **LAG3** | 0.000026 | 9.217 | 8.247 | 0.9693 | 0.1957 | 4.952 | 30.26 | 0.000657 |
| **PD-1** | 0.000126 | 8.244 | 6.526 | 1.718 | 0.3936 | 4.365 | 31.81 | 0.000818 |
| **PDCD1LG2** | 0.003416 | 9.193 | 8.254 | 0.939 | 0.2954 | 3.179 | 29.99 | 0.006622 |
| **PRF1** | 0.014819 | 7.794 | 6.812 | 0.9819 | 0.3811 | 2.576 | 31.88 | 0.018671 |
| **TBX** | 0.000118 | 7.134 | 5.867 | 1.267 | 0.2882 | 4.398 | 31.26 | 0.000818 |
| **TGFb** | 0.012201 | 11.38 | 11.79 | -0.406 | 0.152 | 2.67 | 29.51 | 0.018086 |
| **TNF** | 0.001288 | 8.457 | 7.48 | 0.9775 | 0.2757 | 3.546 | 30.44 | 0.002921 |

**Supplementary Table 2.** Differentially expressed genes with an FDR q value <0.05 between CD3 “high” vs. CD3 “low” canine oral carcinomas.
